# Bibliometric analysis of oncology communication from 1976 to 2025: a focus on narrative medicine

**DOI:** 10.1007/s00520-026-10801-z

**Published:** 2026-05-29

**Authors:** Ismael Traba Outes, Diego Gómez-Carmona, Araceli Galiano-Coronil

**Affiliations:** 1 Faculty of Social Sciences and Communications, Cadiz University, La Asunción University Campus, Avda de La Universidad 5, Jerez de La Frontera, Spain; 2 Instituto Universitario Para El Desarrollo Social Sostenible (INDESS), Jerez de La Frontera, Spain

**Keywords:** Clinical communication, Oncology, Narrative medicine, Bibliometric analysis

## Abstract

Clinical communication in oncology has become a central component of supportive cancer care, with direct implications for symptom management, psychosocial distress, decision-making, caregiver support, survivorship, and palliative transitions. This bibliometric analysis examined 949 documents indexed in the Web of Science Core Collection between 1976 and 2025 in order to map the field’s intellectual structure, publication profile, and thematic development. The study combined PRISMA-guided corpus construction with bibliometric techniques including co-citation analysis, thematic clustering, and strategic mapping. The results show sustained growth across five decades, but also a distributed publication profile, moderate international collaboration, and only partial integration across citation communities. A major transition occurred between 2005 and 2007, when communication began to be framed less as information transfer and more as a therapeutic and relational component of care. Within this structure, narrative medicine remains visible mainly as a conceptual orientation rather than as a consolidated methodological line. Thematic development is stronger in psychosocial, patient-centered, and palliative communication than in digitally mediated, developmentally specific, participatory, or structurally informed approaches. Overall, the findings position oncology communication as an established but still uneven component of supportive care, with practical implications for multidisciplinary teams in nursing, psycho-oncology, social work, and palliative care.

## Introduction

In oncology care, clinical communication is a central axis that directly affects patients’ quality of life, therapeutic adherence, emotional coping, and shared decision-making. The growing complexity of healthcare settings, marked by the technification of diagnosis, the increase in personalized treatments, and the diversity of cultural values, has positioned communication as an instrumental tool and structural component of cancer care. Recent evidence reinforces this centrality: patient-centered communication significantly improves treatment adherence and emotional well-being [46, 61], while ineffective communication increases the risk of high anxiety, unnecessary hospitalizations, and difficulties in decision-making [11, 49]. However, this consolidation has followed an uneven trajectory, articulated around specific protocols that, although effective locally, hinder the construction of integrated knowledge about the relational and narrative dimensions of cancer care.

The formalization of communication training in oncology has progressed through several stages. Early contributions established communication skills as a domain susceptible to systematic instruction, noting their role in reducing patient anxiety and professional burnout [54]. The SPIKES protocol [4] marked a turning point in standardization for delivering bad news, followed by work addressing the complexities of prognostic disclosure [36, 41]. Meta-analytic evidence has confirmed the effectiveness of training programs [9, 19], and international studies have demonstrated their cost-effectiveness [37, 52]. However, implementation remains uneven, only 23.7% of certified cancer centers in Germany offer regular communication training, citing lack of time, resources, and institutional support [48].

The scope of oncology communication has expanded beyond the clinician-patient dyad. Epstein and Street [34] positioned patient-centered communication at the core of practice, emphasizing shared understanding [5] extended this to prognosis communication and the maintenance of realistic hope in palliative contexts. Cancer is increasingly recognized as a family experience: open communication promotes treatment adherence [15], caregiver burden is a clinical concern in its own right [31] and peer support reduces anxiety and depressive symptoms [56, 63]. Health literacy has been reconceptualized as a civic competence that conditions patients’ capacity to engage in care [58], and calls for regulatory protocols integrating emotional support have appeared in several European contexts [14, 66], though adoption remains limited [20]. This evidence reinforces that clinical communication improves adherence, quality of life, and satisfaction [33, 74, 75], while highlighting the distance between knowledge and routine practice.

This evolution in the field of knowledge highlights a conceptual shift that transcends procedural aspects, with consolidation in medical education and in the management of complex chronic conditions [23]. The study by [29] shows the speed of adaptation in health communication during COVID-19, demonstrating that peripheral fields such as digital communication can be quickly reorganized in the face of disruptive phenomena, while established domains evolve gradually. This phenomenon suggests that oncology communication is in an emerging phase. Furthermore, the work of [47] documents the heterogeneity of fields in transition toward disciplinary maturity. Specifically, the authors state that, in oncology communication, there is specialization within some general subfields, but further research is needed in others. The research by [8] highlights editorial dispersion, typical of emerging disciplines. It points to the absence of standardized methodologies to measure its effects, thereby perpetuating its epistemologically ambiguous position within dominant biomedical hierarchies [2]. Health communication encompasses multiple disciplines, such as psycho-oncology, medical ethics, health sociology, and the social sciences, generating significant conceptual richness but also heterogeneity that makes it difficult to articulate a single frame of reference. This situation is marked by the rapid transformation of the clinical environment, driven by phenomena such as the digitization of care, the reconfiguration of healthcare relationships in the wake of COVID-19, and the emergence of artificial intelligence-based technologies [70, 73]. This evidence reveals a critical gap: no bibliometric analysis has specifically mapped how narrative medicine—which, as formalized by [22], operates as a philosophical reframing of the clinical encounter that recognizes patients’ illness narratives as clinically relevant, as a trainable competence enabling professionals to recognize, absorb, and interpret those narratives, and as a set of specific methods including reflective writing and parallel charts—has been integrated into the intellectual architecture of oncological communication. These three dimensions represent a gradient from conceptual presence to operational practice and focusing on them can reveal which has achieved citation visibility—whether narrative medicine has entered the field as a philosophical reference, as an established competency domain, or as a methodological tradition with its own research base.

This is the first bibliometric study to map narrative medicine within oncology communication. Specifically, this study seeks to address the structural, informational, and thematic gaps in scientific production on clinical communication and oncology, as indexed in the Web of Science (WoS), from 1976 to 2025. Using advanced co-occurrence analysis techniques, co-citation networks, and thematic clustering, we will delve into the intellectual architecture to identify emerging themes, key authors, and research gaps. Specifically, the following objectives were established: (a) to study the field of knowledge on clinical communication and oncology through bibliometric analysis of Web of Science; (b) to identify authors, journals, and works that have contributed most frequently, visibly, and impactfully to the construction and scientific development of the field of study; and (c) to discover the main thematic clusters, identifiable conceptual trends in the scientific literature on clinical communication in oncology, and under-explored or emerging areas in order to propose a future research agenda for the discipline.

These objectives led to the exploration of the following key questions:

- Research Question 1: *What type of scientific evidence has been published in the Web of Science database regarding communication interventions in the field of oncology, and how has it evolved?*
- Research Question 2: *Which journals and authors are the most productive and have the most significant scientific impact in the study of communication interventions in oncology, according to the leading academic indicators?*
- Research Question 3: *What thematic clusters and intellectual structures predominate in clinical communication in oncology, and how do narrative approaches position themselves in relation to dominant paradigms, pointing out gaps and emerging lines of research?*

The following sections present the methodology, results, and discussion.

## Methodology

This study is a bibliometric analysis designed to map the intellectual structure and evolution of scientific knowledge on clinical communication in oncology, following the methodological recommendations established by [30] and [76]. The study corpus was limited to the Web of Science Core Collection (WoS), a multidisciplinary database covering more than 250 areas of knowledge and with a selective editorial process that guarantees quality in scientific indexing [24].

### Search strategy and corpus construction

The search equation was constructed using a conceptual block approach, breaking the topic into its fundamental semantic components for subsequent combination using Boolean operators. After a preliminary review of the literature and consultation of specialized thesauri, two main blocks were identified:

- Block A (Oncology domain): Terms related to cancer, neoplasms, and oncology care
- Block B (Clinical communication): Terms related to professional–patient interaction and healthcare communication

#### Initial search

A refined thesaurus was developed by integrating MeSH descriptors, Scopus thesauri, and WoS. The terminology selection was based on the narrative-relational framework defined in the theoretical section [16, 17, 22], including descriptors such as “narrative medicine,” “empathic communication,” “shared decision-making,” and “breaking bad news” to capture the dimension of interpretive co-creation. The Title (TI) field was prioritized over Topic (TS) to maximize semantic precision, following [62]. The final equation combined both fields and document type filters (article and review) and thematic categories, resulting in 979 documents (April 19, 2025).

The result of this first phase is the following search equation:

“(TI=(“cancer” OR “neoplasm*” OR “oncolog*” OR “tumor*” OR “carcinoma”) AND TI=(“communication” OR “health communication” OR “doctor-patient communication” OR “patient-provider communication” OR “physician-patient relations”)) AND TS=(“narrative medicine” OR “empathic communication” OR “health literacy” OR “shared decision-making” OR “risk communication” OR “digital communication” OR “breaking bad news” OR “prognostic disclosure” OR “palliative care” OR “end-of-life care” OR “terminal cancer” OR “advanced cancer” OR “psychosocial care” OR “telemedicine” OR “telehealth” OR “mHealth” OR “patient portal*” OR “online support group*” OR “social media communication” OR “treatment adherence” OR “quality of life”) AND DT=(Article OR Review) NOT WC=(Biochemistry & Molecular Biology) and Proceeding Paper or Early Access or Book Chapters (Exclude - Document Types)”

#### Data cleaning and screening

The cleaning and management operations were carried out using a combination of *Bibliometrix* (R) and its graphical interface *Biblioshiny*, specialized software widely recognized for its ability to analyze metadata, semantic networks, and co-citation structures [3]. The cleaning operations included technical and structural aspects of the corpus (field format, encoding compatibility, and metadata preservation), followed by the application of thematic exclusion criteria.

#### Exclusion criteria

Once the initial corpus had been cleaned, a manual and semi-automated review of titles and abstracts was conducted using clearly defined exclusion criteria. No predefined lists of excluded keywords were established, as the focus was on preserving the field’s semantic diversity. The selection of records was carried out in two successive stages: first, a blind reading of the title and abstract; second, refinement by thematic exclusion, without the need to read the full text, given the semantic clarity of the records. According to [27], the exclusion criteria helped discriminate studies focused exclusively on: aspects of sexuality in contexts unrelated to clinical communication; economic or healthcare-cost analyses; institutional or commercial communication; and non-oncological diseases. This decision allowed us to define precise thematic frameworks to ensure epistemological consistency [12].

These reviews were performed using semi-automated tools implemented using Python in Google Colab, evaluating key terms in titles and thematic fields. The records were imported into EndNote, enabling efficient organization of the corpus prior to analysis in R.

This process was complemented by validation through a cross-check of 10–15% of the total records, which allowed for the detection of residual biases, refinement of the corpus’s thematic consistency, and greater methodological robustness [27, 38].

#### Inclusion phase

The final corpus consisted of 949 documents from the Web of Science database, which served as the basis for this study.

### Lexical curation and terminological standardization

After completing the document screening phase and aiming to optimize the semantic and structural quality of the corpus for bibliometric analysis, a lexical curation process was conducted to standardize terminology in the “Author Keywords” and “Title” fields. This action was not part of the document inclusion or exclusion procedure but instead responded to a subsequent analytical need: to ensure semantic consistency in the analyses of co-occurrence, n-grams, and keyword networks. The standardization of terms is an essential step to avoid conceptual dispersion in semantic maps [67]. Cobo et al. [26] recommend applying cleaning and terminological unification processes to ensure interpretative robustness in the construction of scientific networks.

As a preliminary step to this curation, a preliminary exploratory survey of terms in the aforementioned fields was conducted, which allowed for the identification of lexical redundancies, spelling variants, and equivalent forms (e.g., “shared decision making” vs. “shared-decision-making”), hyphenated structures, acronyms, or inconsistent pluralizations. Similar procedures have been recommended by [67] and [3], who highlight the usefulness of applying preliminary exploratory analyses as a basis for creating synonym dictionaries and terminology standardization files. Based on this survey, a *synonymous file* was created.

### Techniques and metrics for data analysis

R, with the *Bibliometrix* package and its *Biblioshiny* interface [3], was used as the primary tool for bibliometric analysis. These platforms enabled us to apply a set of metrics to evaluate scientific productivity, thematic dynamics, and collaboration networks. To measure the scientific productivity of authors, the total number of publications, their annual evolution, and the h [42], g [32], and m [13] indices were considered. The authorship fractionation technique [60] was also incorporated, which adjusts productivity based on the number of co-authors per article and compares total production with fractionated production.

In terms of the academic impact of the documents, local (within the corpus) and global (total) citations were analyzed, along with other citation indicators, which made it possible to assess the specific relevance of the publications in the area. At the relational level, term co-occurrence analysis [21] was applied and processed using the Leiden algorithm [65] to generate thematic networks and detect communities of authors. Finally, the relative position of the thematic areas was visualized using a thematic map, following the procedure described by [26].

## Results

### Scientific output in clinical communication and oncology

The study focused on 949 documents indexed in the Web of Science Core Collection (WoS) between 1976 and 2025, distributed across 296 journals and with an annual growth rate of 6.79%. These indicators together characterize oncology communication as a growing but structurally uneven field. An expanding knowledge base coexists with persistent editorial dispersion and only partial integration among research communities, with consequences for the differential availability of evidence-based tools across clinical disciplines. The corpus reflects moderate international collaboration (16.33%) and an average document age of 8.52 years, indicating a relatively recent consolidation despite a time frame of approximately 5 decades.

The temporal evolution of the field reveals three clearly differentiated phases. The early exploratory period (1976–1999) began with the work of [50], which analyzed affective communication in patients with advanced cancer and was published in Psychosomatic Medicine. This foundational study, framed within the psychosomatic tradition, addressed the emotional dimensions of the oncological experience, anticipating contemporary approaches focused on relational care. During this stage, scientific production remained marginal, with sporadic publications, lacking thematic coherence and without sustained research programs.

The protocolization phase (2000–2009) marked a critical turning point. The volume of publications increased sevenfold, coinciding with the publication of the SPIKES protocol by [4] for communicating bad news. This standardization signaled a disciplinary shift from intuitive practice to evidence-based communication training. This period consolidated communication as a skill susceptible to systematic instruction, recognizing its impact on treatment adherence, emotional coping, and psychosocial distress management—outcomes that would become central to the supportive care agenda in oncology. The institutionalization of training programs, particularly visible in journals such as Patient Education and Counseling and Psycho-Oncology, laid the methodological foundations for the subsequent expansion of the field, with systematic reviews demonstrating the effectiveness of these programs [39] and the consolidation of patient communication needs as a structural axis of care [40]. The exponential expansion phase (2010–2025) shows sustained growth, with 86 publications in 2024 alone. This acceleration corresponds to the consolidation of patient-centered communication frameworks [34] and narrative medicine approaches [22], which reconceptualize communication as a therapeutic intervention rather than an auxiliary practice. The proliferation of research on quality of life [35], shared decision-making [43], and survivorship reflects a maturation toward models that integrate symptom management, psychosocial support, and caregiver communication [71] that constitute the core of supportive oncology care. At the same time, digitally mediated communication remains only weakly visible in the communication-explicit corpus, despite its increasing relevance in actual oncology care. This pattern suggests that the literature has consolidated more quickly around psychosocial, palliative, survivorship, and caregiver-related communication than around technology-mediated encounters.

**Fig. 1:**
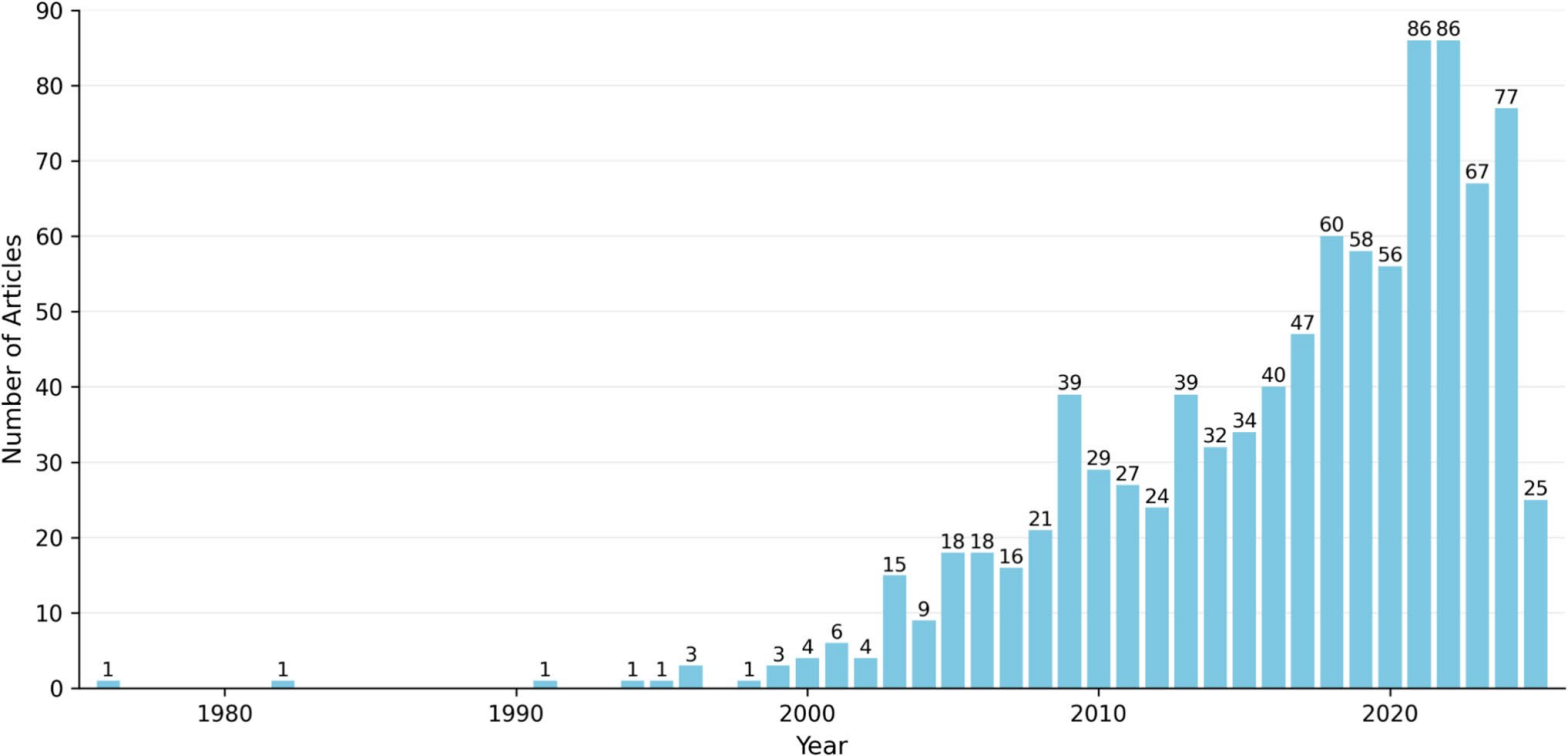
Evolution of scientific output in clinical communication and oncology. Source: Authors with data from Web of Science (2025)

Table 1 and Fig. 1 document these patterns, showing accumulation dynamics typical of fields transitioning from a peripheral to a semi-central position within the health sciences. The trajectory of sustained growth, together with persistent editorial dispersion, indicates a discipline advancing in consolidating its intellectual identity while managing structural tensions between progressive specialization and interdisciplinary dialogue.

**Table 1:** Indicators of the analyzed corpus, Web of Science (1976–2025). Source: Authors (2025)

Indicator	Data
Coverage period	1976–2025
Total number of documents	949
Number of sources (journals)	296
Annual growth rate	6.79
Total number of authors	4,450
Single-author documents	29
Average number of co-authors per document	6.28
Percentage of international co-authorship	16.33
Total number of keywords (DE)	1,684
Total references cited	26,900
Average age of documents (years)	8.52
Average citations per document	26.59

### Sources of scientific publication in clinical communication and oncology

The distribution of scientific output across 296 journals, with only 18 sources exceeding 10 publications (Table 2), shows a low editorial concentration (7.2%), in clear contrast to mature fields, where a higher percentage of output tends to be concentrated in a small core of central journals. *Patient Education and Counseling* leads the ranking with 68 documents (7.2%), followed by *Supportive Care in Cancer* (63) and *Psycho-Oncology* (57). These leading journals correspond to patient education, supportive care and psycho-oncology indicating that the field’s editorial base is distributed across the clinical domains most directly involved in managing symptoms, psychosocial needs, and quality of life throughout the cancer trajectory. The presence of the *Journal of Clinical Oncology* (17 publications, 1.8%) suggests that communication is visible within mainstream oncology, although it does not occupy a dominant editorial position relative to more biomedical priorities. Journals such as *the Journal of Cancer Education* and *the European Journal of Oncology Nursing* highlight the emergence of educational and nursing dimensions. In contrast, generalist platforms such as *BMJ Open* and *PLOS ONE* reflect the field’s dissemination across disciplines.

**Table 2:** Journals with a frequency equal to or greater than 10 in the number of publications in clinical communication and oncology. Source: Authors with data from WoS (2025)

Journal	Number of documents
Patient Education and Counseling	68
Supportive Care in Cancer	63
Psycho-Oncology	57
Journal of Cancer Education	28
Journal of Palliative Medicine	19
Cancer	18
Cancer Nursing	18
European Journal of Cancer Care	18
Health Communication	17
Journal of Clinical Oncology	17
BMJ Open	16
Journal of Pain and Symptom Management	15
Journal of Health Communication	14
European Journal of Oncology Nursing	13
Palliative & Supportive Care	13
Pediatric Blood & Cancer	13
BMC Cancer	11
Cancers	11
PLOS ONE	10

By observing the evolution over time, it is possible to differentiate between established and emerging sources. *Patient Education and Counseling*, *Supportive Care in Cancer,* and *Psycho-Oncology* began their activities in this field before 2000, demonstrating sustained editorial commitment for more than 25 years. In contrast, later additions, such as *the Journal of Cancer Education*—fourth in productivity—exemplify the field’s thematic expansion beyond traditional psycho-oncological frameworks, focusing on pedagogical, training, and professional development concerns. This shift suggests a progressive institutionalization of oncological communication as a cross-cutting competence in health training, beyond its exclusive consideration as a psychosocial variable.

#### Productivity by authors

To provide greater clarity and a more consolidated response, productivity by author was also studied, including both published and fractionalized articles (Table 3).

**Table 3:** Ranking of authors with more than 10 publications in clinical communication and oncology. Source: Authors using data from WoS (2025)

Authors	Articles	Fractionalized articles
Mack, J	22	3.60
Tulsky, J	21	2.69
Bylund, C	18	2.10
Baker, J	16	2.29
Parker, P	14	2.07
Wittenberg, E	13	2.81
Banerjee, S	12	1.24
Shen, M	12	1.62
Brédart, A	11	2.40
Smets, E	11	1.66
Dolbeault, S	10	2.07
Henselmans, I	10	1.46
Pollak, K	10	1.23
Stiefel, F	10	2.13

Analysis of authors’ productivity allows us to distinguish between volume of production and intensity of intellectual leadership. Mack J. (22 articles, 3.60 fractional) and Tulsky J. (21 articles, 2.69 fractional) have collaboration profiles characteristic of large research groups, in which high output mainly reflects the existence of consolidated institutional infrastructures rather than the intensity of individual contributions. In contrast, authors such as Wittenberg E. (13 articles, 2.81 split) and Brédart A. (11 articles, 2.40 split) show more autonomous academic profiles, with high ratios of individual contribution, suggesting leadership in small teams or a greater orientation towards conceptual and methodological innovation.

Subsequently, researchers were ranked by H-index, and the analysis was completed using metrics commonly used in bibliometric studies.

The impact indicators (Table 4) allow us to identify three distinct types of authors. First, authors of structural relevance combine sustained productivity with a high rate of citation accumulation. Tulsky J. (h-index = 14, 1,412 citations distributed across 21 publications, with an average of 67.2 citations per document) exemplifies this profile, maintaining his influence through regular contributions to core debates in the field. Wittenberg E. also shows high productivity (index m = 0.522), indicating constant involvement for more than two decades, rather than occasional interventions of exceptional impact. Secondly, authors with high conceptual authority are identified who accumulate disproportionate volumes of citations relative to their limited output. Baile W. (9 publications, 1,587 citations, 176.3 citations per article) and Arnold R. (9 publications, 1218 citations, 135.3 citations per article) clearly represent this pattern. Their influence stems from seminal works that remain essential references decades after publication. Their relatively modest m indices should not be interpreted as low productivity, as they are, in fact, a reflection of long careers in which the impact is concentrated in paradigmatic contributions with high structural power. Thirdly, potentially emerging authors such as Smets E. (h-index = 9, m-index = 0.643, beginning publication in 2012) and Alexander S. (h-index = 8, with an average of 94.25 citations per article) stand out, showing rapid impact accumulation over relatively short periods. This pattern suggests the consolidation of new lines of research with high potential for future growth, aligned with more current topics such as patient participation, shared decision-making, and the cultural adaptation of communication models.

**Table 4:** Ranking of the 10 authors with the most significant local impact in clinical communication and oncology according to h-index and other indicators. Source: Authors with data from WoS (2025)

Author	H-index	G-index	M-index	Total citations	No. of publications	Start year
Tulsky J	14	21	0.667	1412	21	2005
Wittenberg E	12	13	0.522	288	13	2003
Mack J	11	22	0.55	729	22	2006
Baker J	10	15	0.714	248	16	2012
Bylund C	10	18	0.625	764	18	2010
Parker P	10	14	0.4	884	14	2001
Smets E	9	11	0.643	300	11	2012
Alexander S	8	8	0.4	754	8	2006
Arnold R	8	9	0.381	1218	9	2005
Baile W	8	9	0.32	1587	9	2001

#### Intellectual structure of clinical communication in oncology

**Fig. 2:**
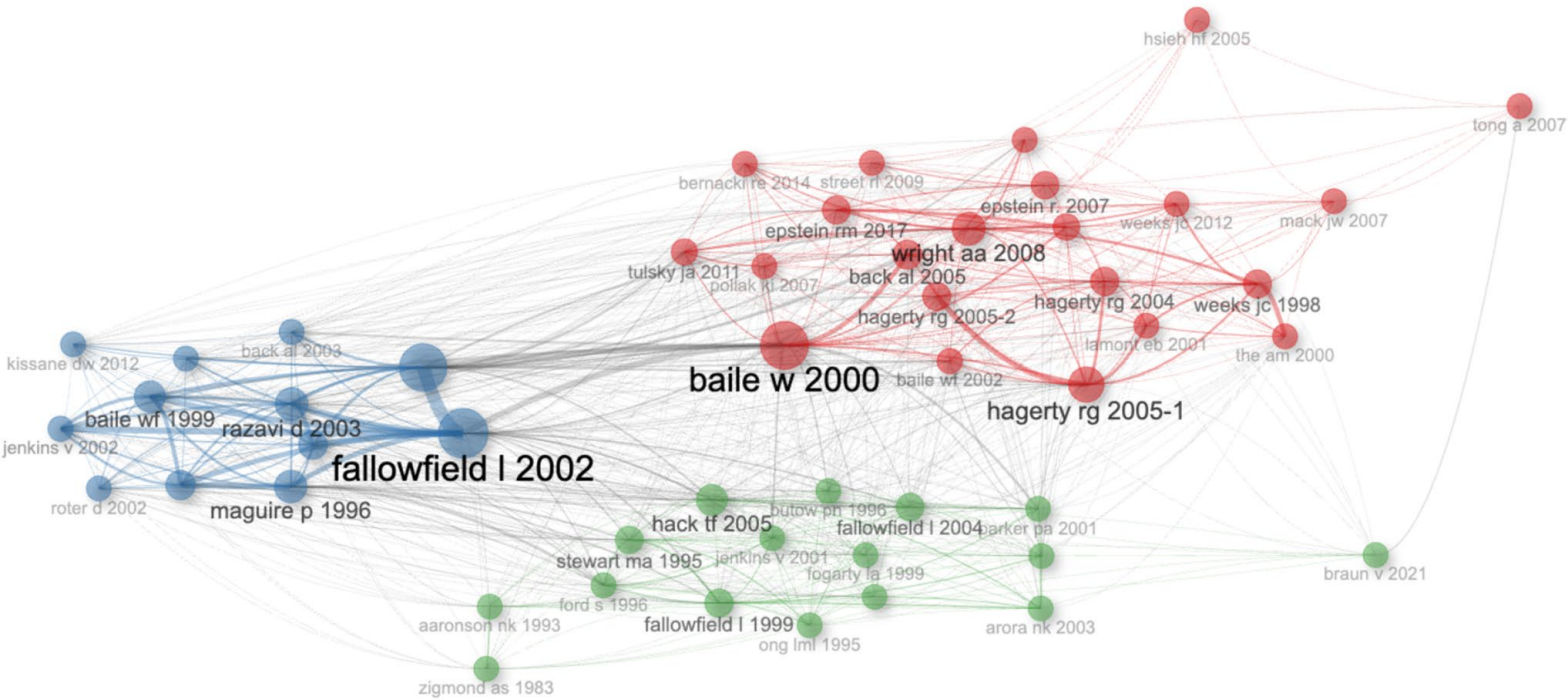
Co-citation structure on clinical communication in oncology. Source: Authors based on data from WoS (2025)

Looking at the co-citation structure (Fig. 2), it is clear that three coexisting paradigms structure the field’s intellectual architecture. The educational-methodological cluster (blue), articulated around Fallowfield L., Baile W., Maguire P., and Roter D., conceives communication as a trainable skill, susceptible to systematic instruction. This paradigm, consolidated through tools such as protocols and various assessment instruments, understands communication as a technical skill acquired through formal pedagogical interventions. Its central position is consistent with the institutionalization of training programs that equip oncology nurses and clinical staff with standardized tools for symptom communication, distress screening, and the delivery of complex diagnostic information. The pragmatic-clinical cluster (red), led by Back A., Tulsky J., Wright A., and Curtis J., focuses on implementing communication in highly complex clinical contexts, especially at the end of life and in palliative care. This community translates evidence into practical guidelines for palliative transitions—communicating prognosis, discussing goals of care, and managing the shift from curative to supportive treatment—competencies essential for palliative care specialists navigating complex clinical conversations. Its high internal cohesion indicates strong collaborative networks and a clear orientation toward transforming clinical practice, where communication becomes a decisive tool for shared decision-making and quality of care. The reflective-humanistic cluster (green), comprising authors such as Hagerty R., Stewart M., and Arora N., articulates biopsychosocial approaches that place the patient as an active subject in the care process, whose expectations, values, and quality of life constitute legitimate clinical outcomes. This tradition can be read as emphasizing the relational dimensions of communication that underpin survivorship care, psychosocial adaptation, and caregiver support, concerns typically addressed by psycho-oncologists and social workers within multidisciplinary teams. Its contribution broadens the field’s analytical framework to include the moral, cultural, and contextual conditions that shape the experience of illness. The network’s density suggests a significant degree of conceptual integration across these three traditions, despite their methodological diversity—with one critical exception: narrative medicine, understood as a clinical competence that enables professionals to recognize, absorb, and interpret patients’ illness narratives [22], and operationalized through methods such as reflective writing and parallel charts, remains peripheral in the co-citation network despite its theoretical recognition. That peripherality takes a different form across each of its three constitutive dimensions. Charon’s reframing of the clinical encounter—the recognition of patients’ illness narratives as clinically meaningful—registers six local citations, substantially below the 26–44 range that defines the field’s most cited works (Table 5), while the term “narrative medicine” appears in two of the 949 documents’ keyword profiles. Narrative competence as a clinical skill, and the specific methods for its development, return no traceable presence across the keyword profiles or co-occurrence structures of the corpus. The peripheral position of narrative medicine therefore takes the form not of a uniform absence but of a differential presence across its three dimensions, whose clinical and theoretical implications are examined in the Discussion.

**Table 5:** Ranking of the 10 articles with the most local citations in clinical communication and oncology. Source: Authors using data from WoS (2025)

Article	Author(s)	Journal	Year	Local Citations	Global citations	LC/GC Ratio (%)	CT Normalized	Normalized CG
*The communication goals and needs of cancer patients: a review*	Hack T. et al	Psycho- Oncology	2005	44	301	14.62	4.60	2.59
*Effect of a Patient-Centered Communication Intervention on Oncologist-Patient Communication, Quality of Life, and Health Care Utilization in Advanced Cancer*	Epstein R. et al	JAMA Oncology	2017	42	313	13.42	10.18	7.53
*Patient-Clinician Communication: American Society of Clinical Oncology Consensus Guideline*	Gilligan T. et al	Journal of Clinical Oncology	2017	41	366	11.20	9.93	8.80
*Approaching Difficult Communication Tasks in Oncology*	Back A. et al	CA: A Cancer Journal for Clinicians	2005	36	441	8.16	3.77	3.80
*Doctor-Patient communication and cancer patients’ quality of life and satisfaction*	Ong L. et al	Patient Education and Counseling	2000	32	327	9.79	2.84	2.42
*Breaking Bad News About Cancer: Patients’ Preferences for Communication*	Parker P. et al	Journal of Clinical Oncology	2001	32	275	11.64	4.27	3.18
*Interacting with cancer patients: the significance of physicians’ communication behavior*	Arora N	Social Science & Medicine	2003	31	461	6.72	6.74	6.97
*Oncologist Communication About Emotion During Visits With Patients With Advanced Cancer*	Pollak K. et al	Journal of Clinical Oncology	2007	27	287	9.41	8.15	6.60
*Communication Skills Training for Oncology Professionals*	Kissane D. et al	Journal of Clinical Oncology	2012	27	217	12.44	10.62	6.71
*Enhancing Communication Between Oncologists and Patients With a Computer-Based Training Program*	Tulsky J. et al	Annals of Internal Medicine	2011	26	208	12.50	6.95	4.06

### Analysis of documents in clinical communication and oncology

The ratio of local to global citations (CL/CG) distinguishes field-specific references from works with broader cross-disciplinary influence.

The most locally cited works (Table 5) constitute the field’s conceptual architecture. The citation patterns reveal a field whose foundational knowledge was produced in two distinct phases—protocolization (2000–2007) and clinical implementation (2010–2017)—with post-2018 production yet to generate comparable foundational contributions.

Global citation patterns (Table 6) reveal a dual structure within the field’s foundational works: specialized tools with high internal impact and transferable frameworks that function as conceptual bridges for adjacent disciplines such as palliative care, medical education, and health services research. The CL/CG ratio distinguishes two functional profiles: works with high ratios (> 12%), such as Kissane et al. (12.44%) and Tulsky et al. (12.50%), serve specialized functions within the field, providing training tools and clinical protocols; works with lower ratios (< 8%), such as Back et al. (8.16%) and Arora (6.72%), operate as conceptual bridges cited across palliative care, medical education, and health services research.

**Table 6:** Ranking of the 10 articles with the most global citations in clinical communication and oncology. Source: Authors using data from WoS (2025)

Article	Author(s)	Journal	Year	Total citations	CT per year	Normalized CT
*Health Literacy and Cancer Communication*	Davis, T. et al	CA: A Cancer Journal for Clinicians	2002	483	20.13	3.29
*Interacting with cancer patients: the significance of physicians’ communication behavior*	Arora, N	Social Science & Medicine	2003	461	20.04	6.97
*Approaching Difficult Communication Tasks in Oncology1*	Back A. et al	CA: A Cancer Journal for Clinicians	2005	441	21.00	3.80
*Patient-Clinician Communication: American Society of Clinical Oncology Consensus Guideline*	Gilligan, T. et al	Journal of Clinical Oncology	2017	366	40.67	8.80
*Doctor-Patient communication and cancer patients’ quality of life and satisfaction*	Ong, L. et al	Patient Education and Counseling	2000	327	12.58	2.42
*Effect of a Patient-Centered Communication Intervention on Oncologist-Patient Communication, Quality of Life, and Health Care Utilization in Advanced Cancer*	Epstein R. et al	JAMA Oncology	2017	313	34.78	7.53
*The communication goals and needs of cancer patients: a review*	Hack T. et al	Psycho-Oncology	2005	301	14.33	2.59
*Oncologist Communication About Emotion During Visits With Patients With Advanced Cancer*	Pollak K. et al	Journal of Clinical Oncology	2007	287	15.11	6.60
*Breaking Bad News About Cancer: Patients’ Preferences for Communication*	Parker P. et al	Journal of Clinical Oncology	2001	275	11.00	3.18
*Enhancing Patient-Provider Communication With the Electronic Self-Report Assessment for Cancer: A Randomized Trial*	Berry D. et al	Journal of Clinical Oncology	2011	267	17.80	5.22

#### Longitudinal patterns of knowledge

Comparing local and global citation rankings reveal three documentary profiles: a consolidated core (Hack, Epstein, Gilligan, Parker) that functions as the field’s conceptual architecture across both metrics; specialized contributions (Kissane, Tulsky, Pollak) whose methodological tools are adopted mainly within the field and transferable frameworks (Davis, Arora) whose influence extends into medical education, health services, and health sociology. The absence of post-2018 publications from either ranking, despite representing approximately 40% of the corpus, confirms that emerging challenges such as digital communication and AI have not yet generated foundational contributions comparable to those of the 2000–2017 period.

### Conceptual structure of clinical communication in oncology

**Fig. 3:**
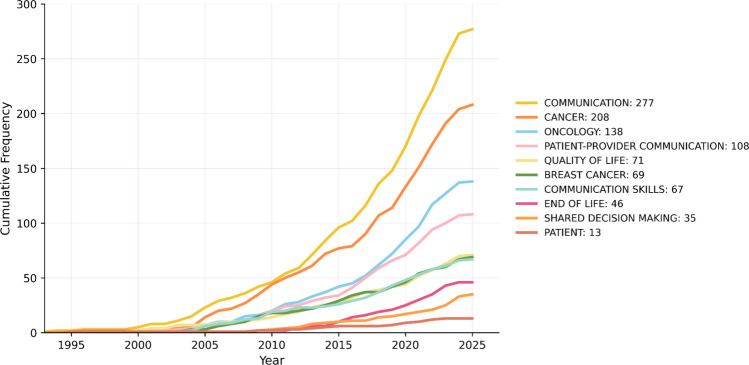
Annual evolution of the 10 most frequent *Author Keywords* in clinical communication and oncology since 1991. *Bibliometrix* with data from WoS (2025)

The chronological evolution of author keywords (Fig. 3) documents a progression from generic categories to clinically operationalized constructs.

The foundational term “Communication” was coined in 1991, establishing the field’s discursive anchor. Its sustained presence over more than three decades demonstrates the continuity of core concerns, while the surrounding vocabulary has transformed, expanded, and grown more complex. The appearance of “Quality of life” in 1995 reflects an ethical-clinical reorientation toward patient-centered outcomes that transcend survival metrics, especially relevant in palliative contexts. For its part, “Patient–provider communication” (1996) marks the transition from general theories of communication to relational frameworks that define the dyadic clinical encounter as the central unit of analysis. The fourteen-year gap until the incorporation of “Shared decision-making” (2005) highlights the gradual transformation from paternalistic models to deliberative approaches, consistent with broader movements toward patient empowerment and the integration of patient values into evidence-based medicine.

This chronological sequence clearly illustrates the conceptual maturation of the field: from abstract constructs of communication (1991) to the relational specification of communicative processes (1996), culminating in participatory decision-making frameworks (2005). The trajectory is consistent with the theoretical evolution identified in co-citation patterns, where early psychosomatic approaches [50] gave way to communication skills training approaches (Baile & Fallowfield) and, subsequently, to patient-centered approaches [34] and narrative medicine [22]. Taken together, this progression reflects an increase in terminological complexity and a shift in the field of knowledge toward more integrative models that recognize clinical communication as a highly relevant relational, ethical, and therapeutic process.

**Fig. 4:**
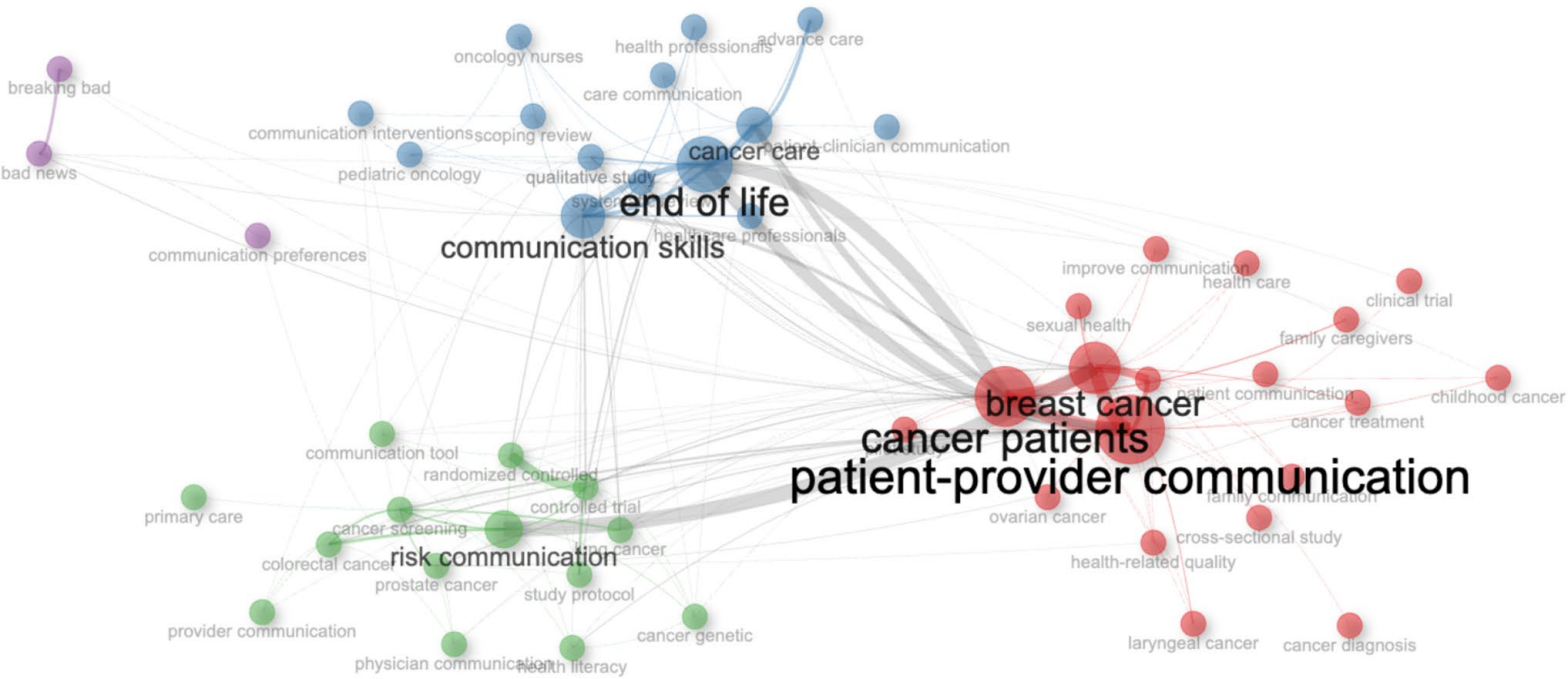
*Title-Bigram* co-occurrence network on clinical communication in oncology. *Bibliometrix* with data from WoS (2025)

The analysis of bigram co-occurrence in titles (Fig. 4) identifies four interconnected semantic fields that structure the domain of study. The relational core (red cluster), composed of terms such as “patient–provider communication,” “breast cancer,” and “cancer patients,” functions as a structural bridge connecting clinical-demographic profiles with communicative modalities. Its centrality indicates that this nexus organizes the relationships between specific disease contexts and communication practices. However, the overrepresentation of breast cancer suggests a concentration of research attention that may reflect broader patterns in oncology research funding and advocacy rather than the communicative complexity of any single cancer type. The training-existential cluster (blue) groups terms such as “end of life,” “communication skills,” and “cancer care,” highlighting the training dimensions concentrated in high-clinical-complexity scenarios. The intense connection with the relational core reveals a remarkable thematic fluidity between general communication and the advanced stages of the disease, articulating a continuity throughout the care trajectories rather than rigid specializations. This integration suggests that end-of-life communication operates as a paradigmatic case for the development of skills, the learning of which is transferable to less acute contexts.

The preventive-predictive cluster (green), focused on “risk communication,” “prostate cancer,” “screening,” and “genetic counseling,” represents communication practices located in the early stages of the care continuum, linked to prevention, early detection, and hereditary risk. Its relative separation from the other clusters indicates limited integration between preventive and therapeutic communication, reflecting broader disconnection between public health and clinical oncology. This disconnect points to missed opportunities to develop longitudinal communication frameworks spanning from prevention to survival. Finally, the ethical-normative satellite (purple cluster), focused on “breaking bad news” and “communication preferences,” is a highly specialized subfield that addresses specific high-risk communicative acts. Its peripheral position reflects specialization around specific protocols and fundamental ethical challenges (autonomy, truthfulness, maintaining hope). The connection with the blue cluster links this specialized domain to broader training agendas, positioning the communication of bad news as an exemplary case for teaching empathic communication in oncology.

### Thematic map

A fundamental part of responding to the third objective lies in interpreting the conceptual structures to identify areas that are underexplored or emerging for future research.

**Fig. 5:**
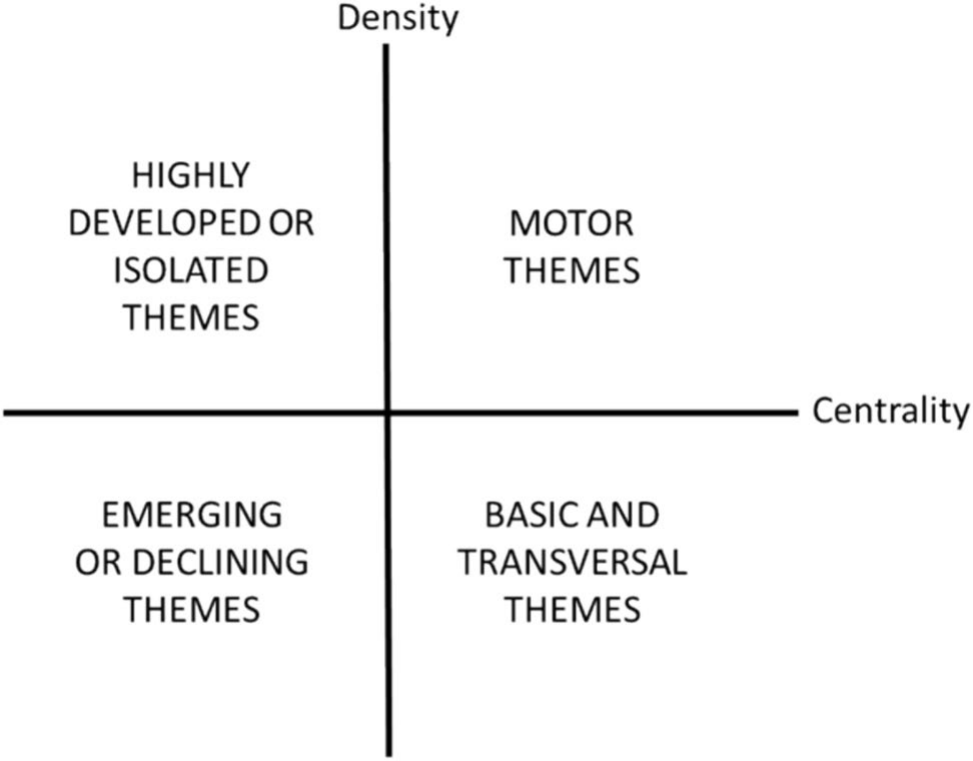
Quadrants of the strategic diagram. Source: Authors based on [26] (2025)

To facilitate interpretation, the results are visualized using a centrality and impact map [26], where clusters of referenced documents are grouped into circles of different colors, whose sizes are adjusted according to their density. The location also allows us to attribute their structural centrality within the field, understood as their degree of connection with other subject areas, and their internal density, which indicates the level of cohesion and conceptual development of the cluster. The following image (Fig. 5) provides visual clarity for understanding the map’s uses.

**Fig. 6:**
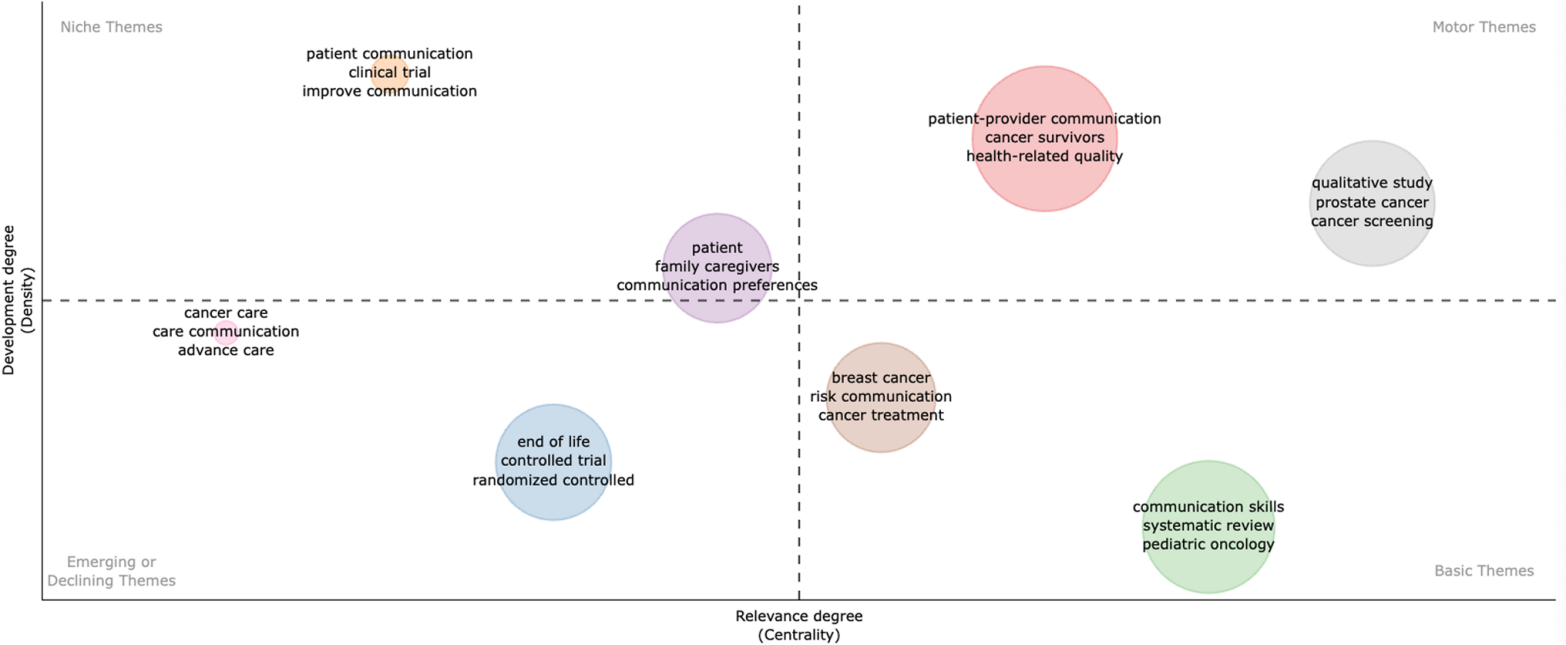
Thematic map based on *Title-Bigram* on clinical communication in oncology. *Bibliometrix* with data from WoS (2025)

Next, we analyze the thematic map (Fig. 6) to identify the basic and driving themes based on their centrality and density, as well as to uncover new potential lines of research.

The driving themes (upper right quadrant) focus on patient–professional communication, cancer survivors, and health-related quality of life, forming the dynamic core of the field. Epstein and Street’s work, Patient-Centered Communication in Cancer Care [34], positions this cluster by considering clinical encounters as collaborative processes that emphasize mutual understanding and shared decision-making. [6] places Social Cognitive Theory within this framework, in which self-efficacy mediates the relationship between communication and health outcomes. Mack and Tulsky’s research on prognosis communication shows how empowering communication styles reinforce patients’ ability to manage uncertainty. Charon’s narrative medicine framework [24] also appears within this cluster.

The basic themes (lower right quadrant) reveal a paradox: communication skills, systematic reviews, and pediatric oncology appear as the foundations of the field, yet lack internal cohesion. Baile, Fallowfield, Jenkins, Kissane, and Back institutionalized training programs following the consolidation of SPIKES [4] and strategies for difficult conversations [36]. [9] provided meta-analytic evidence on the effectiveness of training. [16] acts as a bridge between skills training and clinical ethics by introducing principles of narrative accompaniment, consolidating the field’s turn toward relational dimensions of care. This progression, from technical instruction to communication as a relational competence, reflects a conceptual trajectory with direct implications for how clinical teams are trained across the cancer care continuum. The location of pediatric oncology is particularly problematic, as it is classified as “basic” despite being severely underrepresented (≈ 3% of the corpus), highlighting a disconnect between its strategic importance and the attention it receives to development-specific communication challenges.

Niche topics (upper left quadrant) focus on patient communication, clinical trials, and improvement initiatives, representing methodologically sophisticated intervention research but with limited integration. Hack, Pollak, Clayton, and Tulsky conduct randomized trials to evaluate the effectiveness of training, operationalizing McGuire’s [61] sequential model of persuasion through cognitive stages. [10] demonstrated this through trials with electronic self-reporting systems, while [18, 19] expanded this approach with systematic consultation preparation and training programs that showed measurable improvements. The positioning of this cluster as a niche area reflects its structural isolation from the field’s conceptual core. Methodologically rigorous experimental work on communication training remains largely self-referential, with its advances not yet generating the cross-cluster citation visibility that would indicate clinical practice penetration.

To address the final research objective, four emerging thematic directions were identified in the lower left quadrant, each developing rapidly but without yet generating the citation density needed to enter the field’s conceptual core. Palliative and prognostic communication is gaining empirical grounding. [68] confirm the impact of prognostic communication on palliative clinical outcomes, [64] address its existential dimensions and [28] link goal communication with high-value care practices. Digital and technology-mediated communication accelerated during the COVID-19 pandemic. [44] document digital interventions for patient-professional communication, and [7] formalize tele-oncology protocols based on empathic competencies in technology-mediated environments. Caregiver communication is evolving toward greater structural recognition. [57] demonstrate that integrated geriatric assessment improves communication by incorporating family caregivers, [59] found that patient-caregiver communication predicts burden and preparedness for care and [45] proposed communal frameworks integrating survivors, caregivers, and professionals. Equity-informed and participatory approaches represent an emerging methodological and ethical direction. [1], [53], and [72] document racial, ethnic, and cross-cultural disparities in communication needs and practices, positioning communicative justice as a determinant of health equity. The methodological evolution toward mixed and participatory designs [51] reflects recognition that oncology communication requires methods sensitive to cultural, institutional, and relational particularities.

## Discussion

**Fig. 7:**
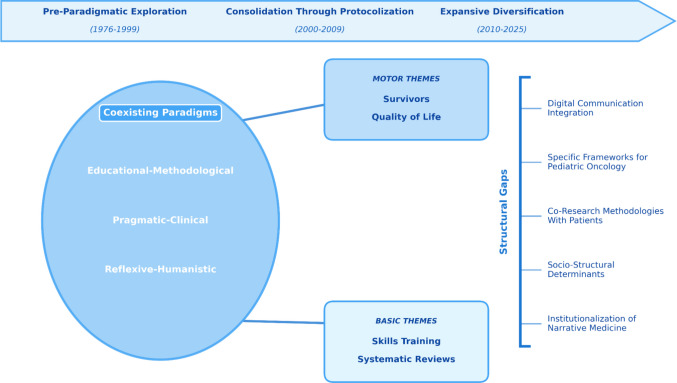
Conceptual model: Intellectual structure and research gaps in oncology communication. Source: Authors (2025)

The study reveals oncology communication as a field in transition toward consolidation. Based on the empirical indicators observed in our dataset, we characterize it as semi-peripheral, a heuristic designation supported by three convergent patterns: low editorial concentration, with no single journal accounting for more than 7.2% of output across 296 sources, limited international collaboration (16.33%) and fragmented co-citation networks, as well as a continued reliance on foundational works from the 2000–2017 period within the co-citation core. As shown in Fig. 7, the intellectual architecture is connected into the educational-methodological (Baile, Fallowfield), pragmatic-clinical (Back, Tulsky), and reflective-humanistic (Charon, Hagerty)—with the relational shift (2005–2007) the milestone that marks the transition from an instrumental transmission of information to approaches that recognize communication as a therapeutic intervention, with narrative medicine as an emerging dimension within reflective approaches. The persistence of protocols and structural gaps shapes the field’s contemporary relevance.

According to the work of [47], oncology communication must address challenges such as communicating diagnoses under uncertainty and discussing prognoses, among others. Furthermore, our work aligns with the findings of [8], who identified weak collaboration networks and journal dispersion, typical features of emerging specializations. In contrast to the work of [29], who documented changes in health communication over just 2 years, our work shows a gradual evolution over the 5 decades observed in oncology communication.

The co-citation analysis shows the SPIKES protocol persisting as a paradigmatic example of the educational approach’s predominance. While standardized frameworks facilitate accessible training, the sustained centrality of protocols alongside the limited visibility of narrative suggests a tension between procedural emphasis and relational depth, a tension widely recognized in the general literature on health communication. Seen across its three constitutive dimensions, narrative medicine’s peripheral position acquires its specific analytical content. While Charon’s philosophical reframing of the clinical encounter circulates as a conceptual reference, neither narrative competence as a clinical skill nor the specific methods for its development appear among the field’s central keyword structures or co-citation clusters. The conceptual recognition of narrative medicine has therefore not been translated into a consolidated research line on its clinical application, leaving the dimension most directly relevant to supportive care teams—the capacity to personalize communication based on each patient’s experience of illness—without a structured evidence base within this literature. Several factors may account for this asymmetry, although they lie beyond what bibliometric data can demonstrate; we present them as interpretive hypotheses rather than empirical findings. Narrative-based research generates outputs less aligned with the publication formats favored by high-impact oncology journals, when such work is published, citation patterns may emerge more slowly, with references functioning as conceptual anchors rather than as inputs to replicable empirical chains, and also the evaluative frameworks governing evidence in oncology tend to prioritize standardized and quantifiable outcomes over interpretive depth—an asymmetry that may shape what enters the corpus as citable evidence and, downstream, what tools become available to clinicians who accompany patients through illness experience. Critical structural gaps become more urgent through comparative analysis. Digital health tools remain underrepresented in the analyzed corpus: Only thirty-one documents (3.27%) contain terms such as *telemedicine*, *telehealth*, *mHealth*, *patient portals*, or *artificial intelligence* in their titles or author keywords, with 25 of these published after 2020. The distribution is revealing. Established modalities, telemedicine, telehealth, patient portals, account for nearly all digital references, whereas terms associated with newer interfaces such as chatbots, video consultations, and remote consultations are entirely absent. This selective pattern suggests that the field has begun to incorporate digital platforms into its research vocabulary but has not yet engaged with the AI-mediated and synchronous modalities that are increasingly reshaping clinical encounters in post-COVID oncology care [7, 44]. The contrast with the exponential growth of AI applications in oncology documented by [73] underscores the widening distance between technological adoption in clinical practice and the communicative research traditions mapped in this study. The scarce presence of pediatric oncology (3% of the corpus), despite its particular evolutionary and family dimensions, is consistent with an adult-centric pattern in the literature. The absence of the patient’s voice as a co-researcher is consistent with broader patterns in health research, where the integration of patient perspectives into study design remains methodologically underdeveloped despite growing recognition of its value. These gaps constitute future challenges that require coordinated investment.

### Theoretical implications

The thematic map configuration carries theoretical implications for how communication research may inform supportive care practice. The driving themes confirm that the field’s most developed research directly serves symptom-related and psychosocial communication, while the basic themes provide methodological infrastructure for training. The absence of digital, pediatric, and socio-structural domains from the consolidated thematic core suggests that the theoretical frameworks currently available to clinical teams are unevenly distributed: protocolized approaches for information delivery at diagnosis and treatment initiation are well supported in the literature, particularly for nursing and medical staff; patient-centered frameworks for prognostic and preference-sensitive decisions in palliative transitions are moderately consolidated and narrative-based approaches for the existential dimensions of survivorship and end-of-life care, where psycho-oncologists and social workers play a central role, remain the least structurally integrated. The limited thematic presence of digital communication, family-centered approaches, and socio-structural determinants indicates that these areas have not yet generated the theoretical density needed to anchor future research agendas within the field.

### Practical implications

The structural gaps identified in this analysis point to areas where clinical teams may lack consolidated research support. Digital communication is a case in point: although oncology teams increasingly conduct tele-consultations and manage symptom reporting through patient portals, the evidence base for maintaining communication quality in these interactions remains limited [7, 44]. Similarly, the underrepresentation of pediatric oncology in the corpus suggests that clinicians working with children and adolescents may have fewer evidence-based tools adapted to developmental stages, parental roles, and sibling needs [53]. The limited presence of communication as an independent outcome variable in clinical trials means that its contribution to adherence, psychosocial adjustment, and quality of life may be underrecognized in the evidence hierarchies that inform resource allocation [35]. Finally, the scant attention to socio-structural determinants—available time, language barriers, burnout, organizational policies—indicates that the conditions enabling effective communication are less well researched than the communication skills themselves, with potential consequences for equity in under-resourced settings [1, 72]. These patterns suggest that supportive care teams—from oncology nurses managing symptom communication to palliative care specialists navigating prognosis discussions to psycho-oncologists and social workers accompanying patients through existential distress—may benefit from a more targeted research investment in the communicative dimensions of their practice.

### Research agenda

Based on the structural gaps identified, six research directions emerge from the analysis. First, the near-absence of digital communication from the thematic core points to the need for frameworks that address communication quality in tele-consultations and remote symptom monitoring. Second, the underrepresentation of pediatric oncology suggests a priority for developing age-appropriate and family-centered communication approaches adapted to developmental stages. Third, the absence of participatory designs in the corpus indicates an opportunity to integrate patients as co-designers of communication interventions and to adopt patient-reported communicative outcomes as primary variables. Fourth, the limited attention to socio-structural determinants—time constraints, burnout, linguistic barriers, organizational policies—signals that research should address the conditions that enable effective communication, not only the skills of individual professionals. Fifth, the peripheral position of narrative medicine points to a need for institutional investment in training and evaluation that bridges the gap between its conceptual recognition and its limited presence in the field’s research core, particularly for the psycho-oncologists and social workers who work with existential distress and meaning-making in survivorship. Sixth, the placement of communication measurement within niche thematic areas suggests that consolidating communication as an independent outcome variable in clinical trials could make its contribution to adherence, quality of life, and survival more visible within the evidence base.

### Limitations

Like any work, this study also has limitations that constrain generalizability and require interpretive caution. The restriction to the Web of Science Core Collection, while ensuring quality control through selective indexing, excludes production hosted in regional repositories (SciELO, LILACS), gray literature (clinical guidelines, institutional protocols), and professional knowledge that circulates through training and professional development networks without formal publication. The Anglo-Saxon linguistic bias inherent in WoS indexing priorities, while allowing access to mainstream knowledge, excludes translations in Spanish, French, Asian, and Global South languages, which could offer alternative conceptualizations shaped by different health systems, communication cultural norms, and specific resource contexts. The representation of digital health communication in the corpus is also shaped by the interaction between our search design and disciplinary publishing conventions. Although digital terms were included in the Topic (TS), the requirement for communication-related terms in the Title (TI), a deliberate design decision, calls for an explicit distinction between the absence of research and artefacts introduced by the search strategy, since the use of communication terms in the title might not accurately reflect communication processes in digital oncology studies. Additionally, the concentration of digital publications in the 2020–2025 period means that the most recent literature may be subject to indexing delays in Web of Science. These factors suggest that the digital underrepresentation documented in the Discussion, while substantive, should be understood as a boundary condition of the present corpus. Bibliometric methodologies capture formal citation patterns within the academic literature but, in some cases, may reflect the actual clinical impact only to a limited extent: highly cited articles may circulate mainly among researchers without influencing healthcare practice, while resources aimed at professionals that are not cited may substantially shape everyday clinical communication. These limitations necessitate complementing bibliometric findings with qualitative research focused on professionals’ perspectives, ethnographic observations of communication practices in clinical contexts, implementation studies documenting the actual adoption of protocols, and participatory approaches that place patients’ experiential knowledge at the center of analysis.

## Conclusions

This study presents a bibliometric mapping of research on clinical communication in oncology based on 949 documents indexed in Web of Science between 1976 and 2025. The results show a field with sustained growth but with an uneven publication and citation profile. The field’s intellectual architecture, structured around three coexisting paradigms, underwent its most significant reorientation between 2005 and 2007, when communication began to be conceptualized more explicitly as a therapeutic intervention rather than merely the transmission of information. Despite this evolution, the analysis identifies significant tensions in the process of consolidating the field. An incomplete institutionalization, sustained mainly by protocolized approaches, persists despite the field’s five-decade growth trajectory. A central finding is the peripheral position of narrative medicine within the field’s intellectual structure: while Charon’s foundational work circulates as a conceptual reference, narrative competence and its associated clinical methods remain outside the field’s co-citation core, limiting the evidence available to supportive care teams for personalizing communication based on patients’ illness experiences.

Thematic analysis also allows us to identify structural gaps with direct implications for the field’s future development. Digital communication is underrepresented in the analyzed corpus, despite its growing relevance in current healthcare settings. Pediatric oncology has a limited presence, which contrasts with the communicative complexity associated with specific family and developmental contexts. Added to this is the scant attention paid to the socio-structural determinants of clinical communication and the absence of the patient as a co-producer of knowledge in the predominant research designs. These findings point to a research agenda aimed at explicitly integrating digital communication and equity, developing developmentally sensitive communication frameworks in pediatric oncology, incorporating participatory methodologies, addressing the structural determinants of clinical practice, and advancing the institutionalization of narrative approaches. Taken together, these findings position clinical communication not merely as a professional skill but as a foundational infrastructure of supportive oncology care—one that spans symptom management, psychosocial screening, caregiver support, palliative transitions, and survivorship—and that requires sustained investment from multidisciplinary teams to fulfill its potential as a determinant of healthcare quality.

## Data Availability

No datasets were generated or analysed during the current study.
